# Low-threshold optically pumped lasing in highly strained germanium nanowires

**DOI:** 10.1038/s41467-017-02026-w

**Published:** 2017-11-29

**Authors:** Shuyu Bao, Daeik Kim, Chibuzo Onwukaeme, Shashank Gupta, Krishna Saraswat, Kwang Hong Lee, Yeji Kim, Dabin Min, Yongduck Jung, Haodong Qiu, Hong Wang, Eugene A. Fitzgerald, Chuan Seng Tan, Donguk Nam

**Affiliations:** 1 School of Electrical and Electronic Engineering, Nanyang Technological University, 50 Nanyang Avenue, 639798 Singapore, Singapore; 2 Singapore-MIT Alliance for Research and Technology (SMART), 1 CREATE Way #09-01/02 CREATE Tower, 138602 Singapore, Singapore; 3 Department of Electronic Engineering, Inha University, Incheon, 402-751 South Korea; 4 Department of Electrical Engineering, Stanford University, Stanford, California 94305 USA

## Abstract

The integration of efficient, miniaturized group IV lasers into CMOS architecture holds the key to the realization of fully functional photonic-integrated circuits. Despite several years of progress, however, all group IV lasers reported to date exhibit impractically high thresholds owing to their unfavourable bandstructures. Highly strained germanium with its fundamentally altered bandstructure has emerged as a potential low-threshold gain medium, but there has yet to be a successful demonstration of lasing from this seemingly promising material system. Here we demonstrate a low-threshold, compact group IV laser that employs a germanium nanowire under a 1.6% uniaxial tensile strain as the gain medium. The amplified material gain in strained germanium can sufficiently overcome optical losses at 83 K, thus allowing the observation of multimode lasing with an optical pumping threshold density of ~3.0 kW cm^−2^. Our demonstration opens new possibilities for group IV lasers for photonic-integrated circuits.

## Abstract (web summary)

Integrating group IV lasing devices into technologically relevant CMOS architectures has proven challenging. Here, the authors demonstrate low-threshold lasing, which is important for potential electronic and photonic circuits, using strained germanium nanowires as the gain material.

## Introduction

Photonic-integrated circuits (PICs) are a key enabler for the miniaturization of various technologies such as light detection and ranging (LIDAR) for autonomous vehicles^1^, bio-chemical sensors^2^ and chip-level optical communications^3^. However, the realization of fully functional PICs is currently limited by the absence of an efficient laser source on silicon (Si)^4, 5^.

Approximately a decade ago, germanium (Ge) began attracting much attention as a potential gain medium for integrated group IV lasers^6^. Unlike Si, the energy difference between the direct Г and indirect L conduction valleys of Ge is only ~140 meV, and therefore it is possible to force some electrons to populate the direct Г conduction valley and to create a population inversion in Ge^6^. By employing heavy n-type doping in ~0.2% biaxially strained Ge to assist with population inversion, optically and electrically pumped Ge-on-Si lasers have been demonstrated^7–9^. However, these lasers suffered from an extremely high lasing threshold current density (~280 kA cm^−2^) that precludes practical integration. It was well known that intrinsic losses from free carriers were a major culprit for this inefficiency^6, 10^, which other researchers identified more specifically as the inter-valence band absorption (IVBA) process from free holes^11, 12^.

During the last few years, researchers have been making relentless efforts to overcome the drawback of indirect bandgap Ge through a variety of techniques. Among those, tin (Sn) alloying into Ge has shown great promise because GeSn alloy with a reasonably high Sn concentration (> 6.5%) can achieve a direct bandgap^13–18^. Recently, a Fabry–Perot type GeSn waveguide with 12.6% Sn demonstrated lasing with an optical pumping threshold density of 325 kW cm^−2^ at 20 K^16^, and a couple of more successful demonstrations have since followed^17, 18^. However, the lasing threshold of those GeSn lasers was still too high to be practical, possibly owing to faster than desired non-radiative recombination processes^16^.

On the other hand, the application of large mechanical tensile strain can also address the challenge of the indirect bandgap in Ge by fundamentally altering the bandstructure^19–25^; the small energy difference between the Г and L conduction valleys can be reduced even further with tensile strain, resulting in an increased material gain^24, 25^. This hypothesis has been supported by numerous theoretical simulations that predict a significant reduction in lasing threshold through tensile strain^10, 26^. With regard to practical realization, several innovative platforms for inducing large mechanical strain have been experimentally demonstrated^19–23^, thus suggesting the possibility of low-threshold group IV lasers.

While lasing from highly strained Ge has been pursued intensively in several recent reports^27–34^, there has been no successful demonstration of optical amplification in such a seemingly promising material system. Most notably, El Kurdi et al.^29, 32^ investigated photoluminescence from direct bandgap Ge under a 1.75% biaxial tensile strain; however, no lasing action was observed even at cryogenic temperatures, possibly owing to a non-uniform strain distribution in the gain medium and/or a poor thermal conduction arising from suspended structures. The authors also performed theoretical calculations on the actual device temperature under the same pump conditions used in their measurements^23^, and found that the temperature rises to 210 K which is significant enough to preclude lasing because of the increased material loss. This temperature-dependent optical characteristic is discussed theoretically and experimentally throughout this study.

Here, we present the observation of low-threshold lasing from Ge nanowires under 1.6% uniaxial tensile strain. With a pulsed optical pumping threshold density of ~3.0 kW cm^−2^ that is two orders of magnitude lower than the state-of-the-art Ge-based lasers^8, 16^, we observe an unambiguous multimode lasing action evidenced by clear threshold behaviours in output power and linewidth as a function of pump power. Theoretical modelling shows that the IVBA, the recently discovered dominant loss factor in Ge^11, 12^, is markedly reduced at cryogenic temperatures^35^ and therefore can be compensated by the amplified material gain in strained Ge, leading to the development of a large optical net gain of ~415 cm^−1^. By presenting unambiguous quantitative evidence of low-threshold lasing action in a fully CMOS-compatible material system, our results pave the way towards a monolithic realization of PICs.

## Results

### Design of strained Ge nanowire lasers

**Fig. 1:**
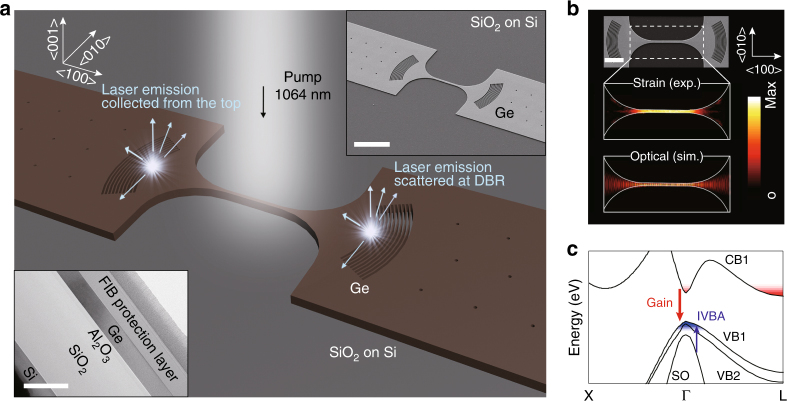
Design of strained Ge nanowire lasers. **a** Schematic illustration of a typical Ge nanowire laser consisting of a strained nanowire surrounded by a pair of distributed Bragg reflectors (DBRs) on the stressing pads. The strained nanowire along the <100> direction is photo-excited with a 1064-nm pulsed laser, and the stimulated emission is collected at a DBR. Top inset: corresponding scanning electron microscope (SEM) image. Scale bar, 10 µm. Bottom inset: cross-sectional transmission electron microscope (TEM) image of the GOI structure. Scale bar, 0.5 µm. **b** Top: top-view SEM image. Scale bar, 5 µm. Middle: 2D strain map measured by Raman spectroscopy showing a highly uniform strain distribution over the entire nanowire gain medium. Bottom: 2D optical field distribution calculated by finite-difference time-domain (FDTD) simulation. A strong spatial overlap between strain and optical fields is achieved in our unique design. The strain and optical field distributions are normalized with respect to the maximum values of each distribution. The maximum strain value is ~1.6%. **c** Calculated bandstructure of 1.6% uniaxial strained Ge. Two major optical processes, gain and inter-valence band absorption (IVBA), are clearly labelled. CB, VB and SO represent conduction band, valence band and split-off band, respectively

The laser structure consists of a Ge nanowire gain medium under 1.6% uniaxial tensile strain along the <100> direction, which is surrounded by two stressing pads containing distributed Bragg reflectors (DBRs) (Fig. 1a). The DBR structure was first designed in ref. ^34^ and further optimized in the present study to minimize optical loss. The strain amplification method first introduced in ref. ^36^ allows for a convenient tuning of the strain level by changing the length of the stressing pads (Supplementary Note 1). The nanowire is optically pumped by a 1064-nm pulsed laser, which allows the emitted photons to strongly oscillate between a pair of DBRs (see Methods). The bottom inset of Fig. 1a shows the cross-sectional transmission electron microscopy (TEM) image of the Ge-on-insulator (GOI) substrate used in the present study (Supplementary Fig. 2).

The unique design of our Ge laser possesses several advantages such as a highly homogeneous strain distribution and an excellent optical mode overlap with the gain medium. Figure 1b illustrates the strong spatial overlap between optical and strain fields in our design. The strain map measured by Raman spectroscopy shows a highly uniform strain distribution of ~1.6% uniaxial tension over the entire nanowire, which in turn allows a homogeneous gain property (see Methods). The contour of an SEM image is superimposed onto the strain map for clarity. The calculated optical field is strongly confined to the nanowire gain medium, thus enabling a large optical confinement factor of ~0.45 according to finite-difference time-domain (FDTD) simulation.

In contrast to the conventional geometry in which the strained Ge gain medium is suspended in air^29–32^, our laser structures hold the strained Ge nanowire in close contact with silicon dioxide (SiO_2_), which provides superior thermal conduction to the air gap while confining optical fields within the Ge layer (Supplementary Fig. 3). While the temperature of the suspended structure quickly increases to 180 K at ~7 kW cm^−2^ similarly to ref. ^32^, our laser structure shows an almost negligible temperature increase with the same pumping conditions (Supplementary Fig. 3d).

Figure 1c presents the calculated bandstructure of Ge under 1.6% uniaxial tensile strain^12^. Two major optical processes, gain and IVBA, are labelled to highlight that gain should overcome IVBA for the occurrence of lasing. The large mechanical strain not only reduces the energy difference between the Г and L conduction valleys but also lifts the degeneracy of the top two valence bands (VB1 and VB2).

### Lasing characteristics in strained Ge nanowire

**Fig. 2:**
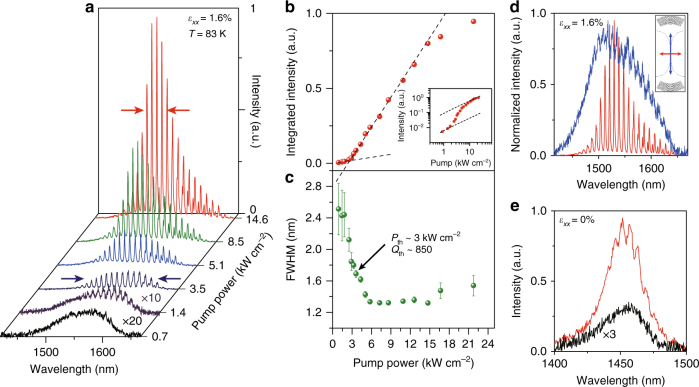
Lasing characteristics from strained Ge nanowires at 83 K. **a** Power-dependent photoluminescence spectra of a 1.6%-strained Ge nanowire with distributed Bragg reflectors (DBRs) showing a gradual transition from broad spontaneous emission to multimode lasing oscillation (threshold, 3.0 kW cm^−2^). The first and second spectra are multiplied by a factor of 20 and 10, respectively, for clarity. The arrows indicate an emission bandwidth of ~150 nm near the threshold (blue) and of < 50 nm in the lasing regime (red). **b** Integrated photoluminescence intensity vs. optical pump power. The black dashed lines represent the linear fit to the experimental data indicating a clear threshold knee behaviour. Inset: corresponding double-logarithmic plot showing nonlinear response to pump power represented by an S-shaped curve. **c** The linewidth evolution of the lasing mode at 1530 nm as a function of pump power. The linewidth narrows from ~2.5 to ~1.3 nm. The error bars are generated by fitting the experimental data to Lorentzian functions. **d** Normalized polarization-dependent spectra collected at 14.6 kW cm^−2^, showing a highly anisotropic gain property of strained Ge nanowires. The emission polarized parallel to the strain axis (blue) does not show optical amplification. **e** Photoluminescence spectra of the unstrained structure taken at 0.7 kW cm^−2^ (black) and 14.6 kW cm^−2^ (red) pump powers, showing no lasing action. The spectrum for the pump power of 0.7 kW cm^−2^ is multiplied by a factor of 3

We successfully observe multimode lasing by optically exciting a 1.6%-strained Ge nanowire integrated with DBRs (Fig. 1a) at a low temperature of 83 K (see Supplementary Note 4 for the cavity design). Figure 2a presents the emission spectra from the laser structure at different pump powers. A linear polarizer inclined perpendicular to the nanowire axis is placed in front of the spectrometer to investigate polarization-dependent lasing behaviour.

At a low pump power of 0.7 kW cm^−2^, the emission shows broad spontaneous emission which consists of two overlapping peaks arising from optical transitions of Γ-VB1 and Γ-VB2^28, 37^. At an increased pump power of 1.4 kW cm^−2^, visible longitudinal cavity modes owing to the presence of DBRs emerge on the long wavelength side as the optical loss is gradually compensated by the material gain. Although such cavity modes near the band edge from highly strained Ge have been already observed numerous times^28–34^, all the previous reports showed the apparent intensity saturation and linewidth broadening of cavity modes with pump powers possibly owing to insufficient gain at low strain^31, 33^, poor optical cavity^28, 29^ and/or unsatisfactory thermal management^32, 34^.

In the present study, on the other hand, the cavity modes near the band edge attain a superlinear increase in intensity along with a significant linewidth reduction as the pump power is increased to 3.5 kW cm^−2^. This phenomenon clearly presents the evidence of optical amplification in highly strained Ge gain medium. Interestingly, the spontaneous emission well above the band edge (<1550 nm) also collapses into sharp cavity modes, thus suggesting that the entire emission band is close to the transparency condition. The full-width at half-maximum (FWHM) of the emission band at this pump power is ~150 nm (marked by two blue arrows). The mode spacing is measured to be ~8 nm, which is consistent with FDTD simulation (Supplementary Fig. 4). Optical simulations also confirm that a fundamental transverse mode can be confined within a Ge nanowire with cross-sectional dimensions of 700 nm × 220 nm (width × height) (Supplementary Fig. 4b). It is worth mentioning that the occurrence of amplified cavity modes over the whole emission band is in stark contrast to conventional lasing materials for which cavity modes are generally observed only within a relatively narrow gain bandwidth. This unique behaviour in highly strained Ge will be explained in the following gain modelling section.

At higher pump powers of 5.1, 8.5, and 14.6 kW cm^−2^, only the modes near 1530 nm continue growing rapidly in intensity as they enter the lasing regime while the modes near the band edge (>1600 nm) appear to saturate. At the highest pump power of 14.6 kW cm^−2^, the cavity modes near 1530 nm obtain >20× higher intensity than the background spontaneous emission, and exhibit a well-defined bell-shaped curve following the net gain spectrum with the FWHM bandwidth of < 50 nm while the bandwidth of the background emission is ~150 nm. The large bandwidths of both spontaneous emission^28, 32, 37^ and net gain^38^ have been previously observed in highly strained Ge, and can be attributed to the unusually large amount of strain-induced valence band splitting (~72 meV for 1.6% uniaxial strain). The FWHM of a single-cavity mode at 1530 nm is determined to be ~1.3 nm by fitting to an individual Lorentzian function (Supplementary Note 5), corresponding to a *Q*-factor of >1100.

Lasing action can also be quantitatively evidenced by the nonlinear behaviour of the integrated output intensity within the gain bandwidth as a function of pump power (Fig. 2b). The output intensity shows a superlinear growth near the threshold pump power (~3.0 kW cm^−2^) followed by a linear growth in the lasing regime, which clearly indicates a distinctive lasing action. The corresponding double-logarithmic plot of the output intensity dependence on the pump power (Fig. 2b, inset) manifests a typical nonlinear threshold behaviour. By employing the multimode laser rate equation to fit our experimental data, we obtain a spontaneous emission factor, *β*, of ~0.08 (Supplementary Note 6). Figure 2c illustrates the linewidth evolution of the highest lasing mode at 1530 nm as a function of pump power. The linewidth reduces from ~2.5 nm to ~1.3 nm above the threshold pump power. The linewidth and the corresponding *Q*-factor at the threshold pump power of ~3.0 kW cm^−2^ is ~1.8 nm and *Q* ~850, respectively, assuming the effective index of 3.2 which is calculated from FDTD simulations. This simulated effective index is also consistent with the reported value in a recent work presenting strained Ge laser structure^32^. The threshold gain is estimated to be 336 cm^−1^ with the simulated optical confinement factor of 0.45. Above 15 kW cm^−2^, the output intensity starts saturating while the linewidth broadens, which can be attributed to the sample heating. This challenge may be overcome by enhancing the thermal conductivity of the Ge nanowire structure by replacing the underlying insulating layer with a more thermally conductive material such as silicon nitride (Si_3_N_4_) and aluminium oxide (Al_2_O_3_).

The polarization dependence of the laser emission is also investigated as shown in Fig. 2d. The emission polarized parallel to the nanowire axis (blue) does not show evidence of optical amplification, manifesting a strong anisotropic gain property caused by uniaxial tensile strain. Figure 2e presents the emission spectra from an unstrained Ge nanowire for 0.7 kW cm^−2^ (black) and 14.6 kW cm^−2^ (red). The emission spectrum at 0.7 kW cm^−2^ is broad and does not exhibit clear cavity resonances, similarly to the spectrum from the 1.6%-strained Ge nanowire under low pumping (black curve of Fig. 2a). While the cavity resonances from the 1.6%-strained nanowire grow rapidly and dominate the background emission at higher pump powers due to the presence of optical net gain (red curve of Fig. 2a), the unstrained nanowire shows only a slight modulation of the emission spectrum at 14.6 kW cm^−2^, resulting in the dominant background emission over the cavity resonances. Further characterization on Ge nanowires with lower levels of tensile strain (0.6 and 1.2%) are also performed (Supplementary Note 7). Similarly to the unstrained nanowire, 0.6%- and 1.2%-strained nanowires do not show any lasing behaviour due to the insufficient strain in Ge, resulting in the absence of optical net gain (Supplementary Fig. 7). These emission features of the Ge nanowires with various strain levels clearly present the significance of tensile strain in achieving optical net gain and lasing from Ge material systems.

### Theoretical modelling for gain and loss in strained Ge

Theoretical modelling is performed for a comprehensive understanding of the gain and loss dynamics in the strained Ge material system. We use the empirical pseudopotential method (EPM) for computing the bandstructure of highly strained Ge, and numerical analysis is employed to calculate the material gain via Fermi’s golden rule^12^ (Supplementary Note 8). For IVBA, we use experimentally extracted absorption cross-sections for room temperature^11, 20^ and cryogenic temperature^35^. The loss is the sum of free electron absorption (FEA) and IVBA, and IVBA is the dominant factor as previously discovered in refs. ^11, 12^ (Supplementary Fig. 8a). At 7 × 10^19^ cm^−3^ carrier density, IVBA is ~214 cm^−1^ whereas FEA is ~96 cm^−1^ for the experimental gain peak wavelength of ~1530 nm, thus making IVBA the major loss mechanism.

**Fig. 3:**
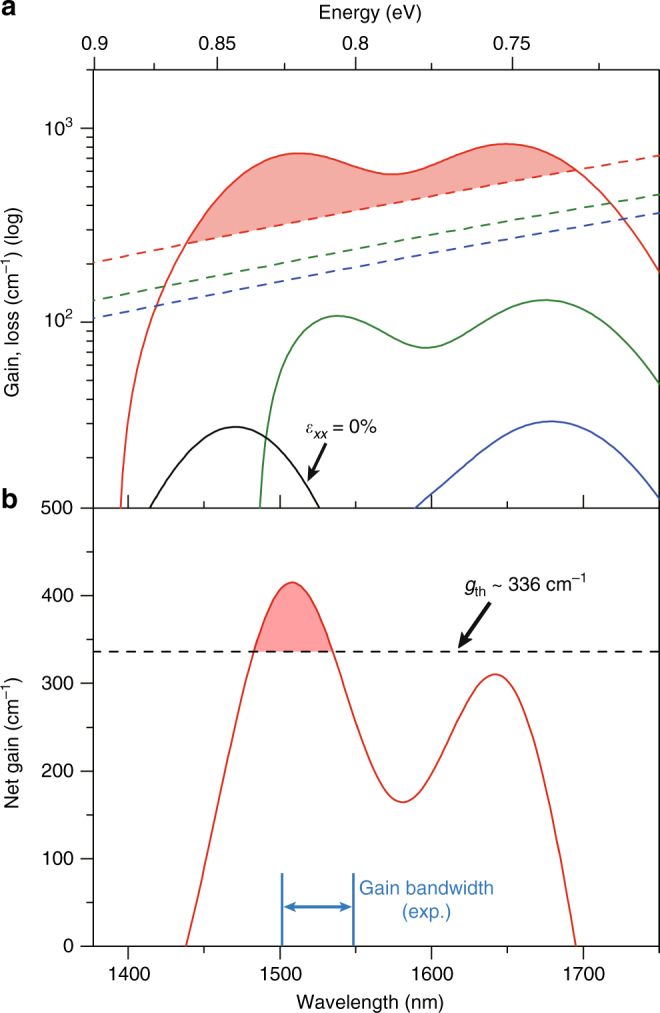
Theoretical modelling for gain and loss in strained Ge at 83 K. **a** Calculated gain (solid line) and loss (dashed line) for 1.6% strained Ge at injection densities of 4 × 10^19^ cm^−3^ (blue), 5 × 10^19^ cm^−3^ (green) and 8 × 10^19^ cm^−3^ (red). The peak optical net gain is ~415 cm^−1^ at ~1510 nm. The gain for unstrained Ge at an injection density of 8 × 10^19^ cm^−3^ (black solid line) is overwhelmed by the loss. **b** Calculated net gain spectrum for an injection density of 8 × 10^19^ cm^−3^. The gain band centred at ~1510 nm only surmounts the experimental threshold gain of 336 cm^−1^. The measured gain bandwidth is also presented as a blue arrow

Figure 3a presents the calculated material gain with reverse sign (solid lines) and loss (dashed lines) at 83 K for three different carrier injection densities of 4 × 10^19^ cm^−3^ (blue), 5 × 10^19^ cm^−3^ (green) and 8 × 10^19^ cm^−3^ (red). At an injection density of 4 × 10^19^ cm^−3^, the material gain attains its peak intensity above 1600 nm, which corresponds to the optical transition between the Γ conduction valley and the top valence band, VB1. As the injection density is increased to 5 × 10^19^ cm^−3^, a large number of holes start filling up the second valence band, VB2, resulting in the emergence of another gain peak below 1600 nm. The significantly increased gain bandwidth extends down to ~1500 nm, thus explaining the experimental observation of cavity modes within such a large spectral range for the spectrum taken at 3.5 kW cm^−2^ in Fig. 2a. The gain continues increasing at an injection density of 8 × 10^19^ cm^−3^ and allows the achievement of a large optical net gain of ~415 cm^−1^ at ~1510 nm. The gain for unstrained Ge at an injection density of 8 × 10^19^ cm^−3^ (black solid line) is overwhelmed by the loss for the same injection density (red dashed line), highlighting the significance of tensile strain for obtaining optical net gain in Ge.

Figure 3b shows the net gain spectrum obtained by subtracting the loss from the gain for an injection density of 8 × 10^19^ cm^−3^. Although there exist two gain bands associated with optical transitions of Γ-VB1 and Γ-VB2, the gain band centred at ~1510 nm only surpasses the experimental threshold gain of 336 cm^−1^. The measured gain bandwidth is also presented as a blue arrow, which is in reasonable agreement with the theoretical gain bandwidth. The slight discrepancy between the theoretical and experimental gain bandwidths may be ascribed to the uncertainty in bandstructure calculation for such highly strained Ge.

### Temperature-dependent emission characteristics

**Fig. 4:**
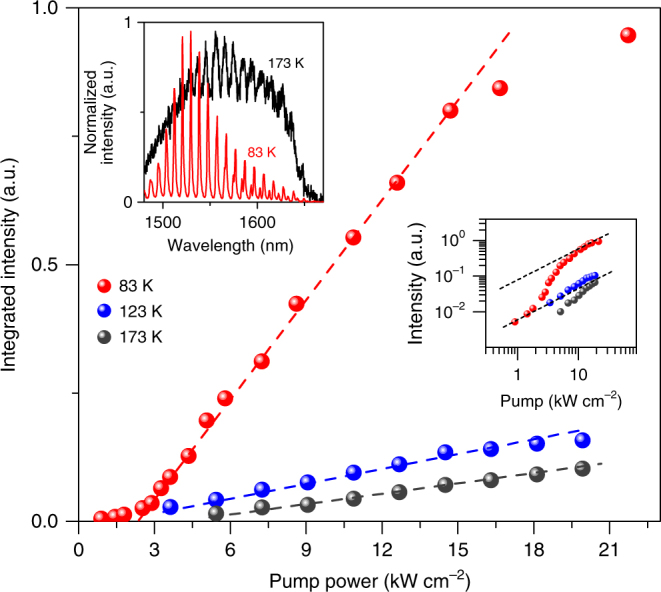
Temperature-dependent emission characteristics. Integrated output intensity vs. pump power of a 1.6% strained structure at temperatures of 83 K (red), 123 K (blue) and 173 K (black). While the data set for 83 K manifests a nonlinear lasing behaviour, no superlinear output increase is clearly observed for 123 K and 173 K. Right inset: corresponding double-logarithmic plot for 3 temperatures. Left inset: normalized emission spectra collected at 83 K (red) and 173 K (black) under a pump power of 14.6 kW cm^−2^. In contrast to the dominant lasing modes for 83 K, the observed cavity modes for 173 K are highly suppressed due to the absence of optical amplification

Figure 4 presents the integrated output intensity of the 1.6%-strained Ge nanowire as a function of the pump power at different temperatures of 83, 123 and 173 K. While at 83 K, optical amplification enables the output intensity to grow superlinearly near the threshold, the data sets for 123 and 173 K do not show such a clear nonlinear threshold behaviour. It should be noted that a clear saturation of the output intensity for 123 and 173 K was observed at higher pump powers owing to the absence of optical net gain. The inset shows the normalized emission spectra taken at 83 K (red) and 173 K (black) at a pump power of 14.6 kW cm^−2^. In contrast to the spectrum for 83 K showing the dominant lasing modes over the background spontaneous emission, the cavity modes are highly suppressed at 173 K and do not show a superlinear growth in intensity at higher pump powers, which displays a strong temperature dependence of gain characteristics in strained Ge. The gain and loss analysis for 300 K shows that optical net gain is not achievable in 1.6%-strained Ge owing to a substantial IVBA, thus highlighting the significant role of IVBA in realizing Ge lasers (Supplementary Fig. 8b).

Recent theoretical studies predicted that room-temperature lasing can be achieved from >3% strained Ge because of the further amplified material gain^12, 20^. Since higher than 3% uniaxial tensile strain has been already achieved in Ge nanostructures^20, 23^, we anticipate that the further development of our laser structure will certainly enable us to obtain such highly strained Ge gain media coupled with high-*Q* optical cavity for the realization of room-temperature Ge lasers.

## Discussion

In summary, we have demonstrated lasing from highly strained Ge nanowires. Our nanowire laser design secures robust mechanical, optical, and thermal properties, which played major roles in enabling low-threshold lasing from highly strained Ge. Excitation-, temperature-, and polarization-dependent optical characterizations showed the achievement of pulsed lasing at 83 K with an optical pumping threshold density of ~3.0 kW cm^−2^, the lowest value among group IV lasers reported to date^7–9, 16–18^. Room temperature lasing could be achieved in our architecture, but with a higher level of tensile strain that is within reach^12, 20, 23^. We believe that single mode operation can be realized by further optimizing the cavity through the use of shorter optical cavities or employing distributed feedback (DFB) gratings. The realization of a low threshold, electrically pumped Ge laser is also possible by using a lateral p–i–n junction^39^. In addition, the ability to tune the strain conveniently by a conventional lithography^20, 21, 36^ should enable the creation of multiple lasers operating at different spectral ranges on a single chip, which is crucial for wavelength division multiplexed optical interconnects. Our demonstration of a low-threshold, highly strained Ge nanowire laser markedly narrows the gap between conventional III–V lasers and their group IV counterparts, thus opening up new avenues for PICs.

## Methods

### Device fabrication

The GOI substrate used in the present work is made via hetero-epitaxy, wafer bonding, grinding, chemical-mechanical polishing (CMP), etc. Detailed explanations along with figures showing step-by-step fabrication processes are provided in Supplementary Note 2. The Ge nanowire laser structures are fabricated via electron-beam lithography, reactive ion etching, wet chemical etching, etc. Further details on the laser fabrication are also available in Supplementary Note 2.

### Raman spectroscopy for strain measurement

A micro-Raman spectroscopy system with a 532-nm laser source was used to measure the Raman peak shift of a strained Ge laser structure. By using Lorenz fit of the peak shift with a uniaxial strain-shift coefficient of 152 cm^−1^, the strain value can be derived from the Raman measurement. Raman mapping was conducted to measure the strain distribution over the laser structure. The piezo stage step size was set to 200 nm during the mapping.

### Photoluminescence spectroscopy for laser characterization

For photoluminescence spectroscopy, a 1064-nm pulsed laser was focused using a ×50 magnification lens. The pulse duration and repetition period were 20 and 100 ns (duty cycle of 20%), respectively, to minimize heating. All pump power values refer to the peak laser intensity considering 20% duty cycle. The spot size was set to ~15 µm to uniformly illuminate 8 µm long nanowires. The laser emission is scattered in a random direction at the DBR mirrors and a fraction of the scattered emission propagating towards the top surface is collected via the same ×50 magnification lens which is used to focus the pump laser (Fig. 1a). The scattering location of the laser emission is confirmed to be right at the first air trench of the DBR mirrors via FDTD simulations. A cooled InGaAs 1D-array detector with the cut-off at 1.7 µm is used for data collection.

### Data availability

The data that support the findings of this work are available from the corresponding author upon request.

## Electronic supplementary material

Supplementary Information
