# Supplementary material for: Low-threshold optically pumped lasing in highly strained germanium nanowires
Source: Nat Commun. 2017 Nov 29;8:1845. doi: 10.1038/s41467-017-02026-w (PMC5705600; doi:10.1038/s41467-017-02026-w)
Supplement: Supplementary file 1 — Supplementary Information [file 41467_2017_2026_MOESM1_ESM.pdf]

Supplementary Note 1: Geometrically tunable strain

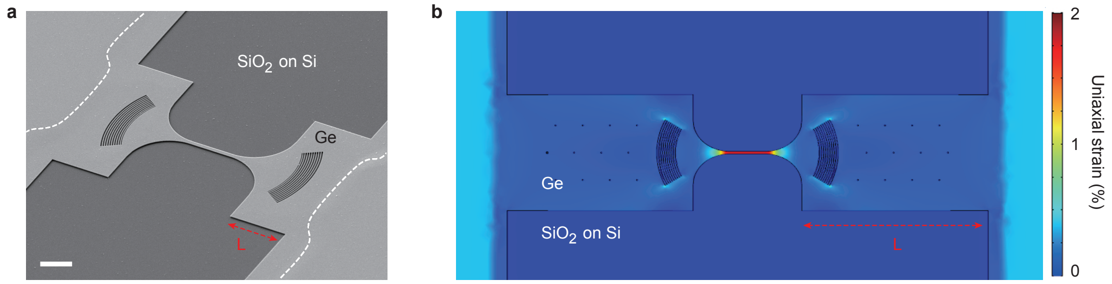

Supplementary Figure 1 | Geometrically tunable strain. a, Scanning electron microscope (SEM) image highlighting the length of the undercut region, *L*, for tuning the level of strain in germanium (Ge) nanowires. Scale bar, 5 µm. **b, Finite-element method (**FEM) mechanical simulation showing a highly uniform strain distribution in the Ge nanowire.

In our design, we employ the strain amplification technique first introduced in ref 1 and integrate an optical cavity in the stressing pads to provide optical feedback to the gain medium. Supplementary Figure 1a shows a scanning electron microscope (SEM) image of an entire geometry highlighting the undercut length, *L*, which is used to adjust the level of strain by a conventional lithography. It is worth mentioning that by having multiple structures with different *L* on a single chip, one can achieve a number of lasers operating at different spectral ranges without complicated material growth such as for compound semiconductor lasers for which the composition of materials determines the operating wavelengths of lasers. Supplementary Figure 1b presents a simulated 2D strain distribution by finite-element method (FEM) mechanical simulation. In the simulation, a pre-existing film strain is set to be 0.2%. As a result of strain redistribution in the undercut structure, an amplified strain of ~2% exists within the entire gain medium uniformly. This allows us to achieve an extremely homogeneous gain medium which also plays an important role in enabling the lasing action in our highly strained germanium (Ge) lasers.

Supplementary Note 2: Fabrication process of substrates and laser structures

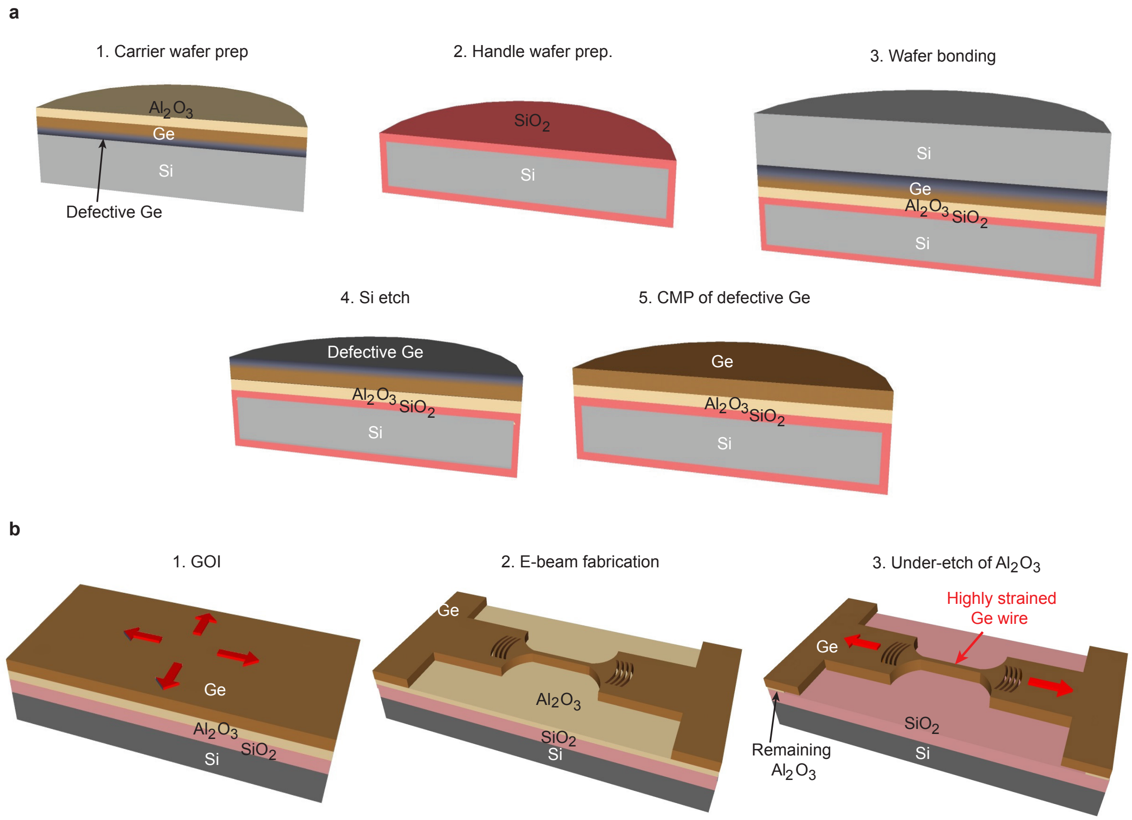

Supplementary Figure 2 | Fabrication process. a, Schematics of the germanium-on-insulator (GOI) substrate fabrication process. **b,** Schematics of the laser structure fabrication process.

Our germanium-on-insulator (GOI) substrate for the germanium (Ge) nanowire laser was made via epitaxy and wafer bonding (Supplementary Fig. 2a), and the laser structure was then fabricated on GOI by lithography and etching processes (Supplementary Fig. 2b). A bulk Ge layer is directly grown on an 8-inch silicon (Si) (100) substrate using Aixtron metal-organic chemical vapor deposition (MOCVD) reactor. A low-temperature/high-temperature (LT/HT) two-step growth mode is used to grow a 50-nm low-temperature (LT) Ge seed layer at 400 ˚C with heavy arsenic (As) doping and a 2.2-µm high-temperature (HT) Ge layer at 650 ˚C[2]. During the HT growth, phosphorus (P) doping is introduced to the top 900-nm Ge layer to enable a uniform doping profile with a target P-doping concentration of ~ 6×10^{18} cm^{-3}. Additionally, thermal cycling is applied to improve the epi-layer quality, leading to a threading dislocation density (TDD) of ~ 4.6×10^{6} cm^{-2}. After removing the top 100-nm Ge layer by chemical mechanical polishing

(CMP) step for a smooth surface for bonding, a 50-nm Al_2O_3 sacrificial layer is deposited on the Ge surface by atomic layer deposition (ALD). Then the Ge-on-Si wafer is directly bonded at room temperature to an 8-inch Si (100) handle wafer with a 1-µm thick thermal oxide (SiO_2) layer, followed by a post-bonding annealing at 300 ˚C for 3 hours to enhance the bonding strength. After removing the carrier Si by grinding and selective chemical etching in Tetramethylammonium hydroxide (TMAH), the Ge layer is transferred to the handle wafer, which forms a GOI substrate. Then the Ge layer of the GOI is thinned down by CMP to the desired thickness of 220 nm with a surface roughness of < 0.2 nm. The 8-inch GOI wafer is diced into 1 × 1 cm^2 pieces for device fabrication. The Ge nanowire laser structure is defined by the electron-beam lithography (EBL), and its pattern is transferred to the Ge layer by reactive etching (RIE) using Cl_2 gas. The dry etch stops at the Al_2O_3 layer, and the sample is wet etched in 30% KOH solvent to selectively remove the sacrificial Al_2O_3 layer forming the undercut structure. The releasing process causes the strain redistribution and amplifies the tensile strain in the nanowire[3,4]. Then the sample is dimmed in 100% IPA. We finalize the laser fabrication by contact drying on a hotplate, which allows the nanowire to be in contact with the SiO_2 layer rather than suspended in air, resulting in a better heat conduction in our device.

Supplementary Note 3: Stiction for heat conduction

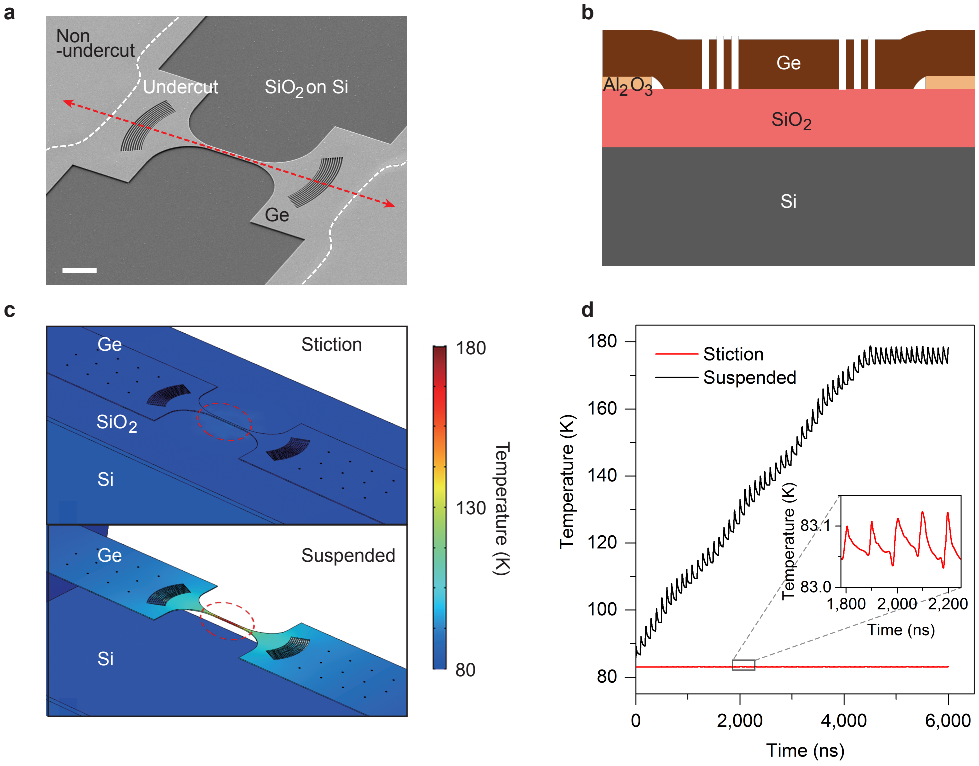

Supplementary Figure 3 | Stiction for heat conduction and thermal simulation of laser structures. a, Scanning electron microscopy (SEM) image of a typical laser structure highlighting the undercut region. Scale bar, 5 µm. **b,** Cross-sectional schematic along the red dashed line of panel a. The germanium (Ge) nanowire gain medium is in close contact with the underlying silicon dioxide (SiO_2). **c,** Stationary finite element method (FEM) thermal simulation for temperature distribution in the stiction and suspended structures under a pulsed optical pumping of ~7 kW cm^{-2} with a spot size of 15 µm (red circle region). **d,** Time-dependent FEM thermal simulation for temperature variation as a function of time for the stiction and suspended structures under the same condition as Supplementary Fig. 3c.

In our design, we intentionally bring the germanium (Ge) layer into contact with the underlying silicon dioxide (SiO_2) layer during the fabrication process (Supplementary Note 2). While the SiO_2 layer can effectively confine the optical mode within the Ge layer owing to a large refractive index difference between Ge and SiO_2, the heat accumulation by optical pumping can be significantly minimized in our architecture since the SiO_2 layer provides additional heat conducting paths towards the thick silicon

(Si) substrate.

An SEM image of the finalized Ge laser structure in Supplementary Fig. 3a clearly shows the boundary between the non-undercut and undercut regions. The undercut region is stuck onto the underlying SiO_2 layer, which allows superior heat conduction compared to the air gap employed in most other strained Ge resonator structures[5–7]. Supplementary Figure 3b presents a cross-sectional schematic along the red dashed line in Supplementary Fig. 3a to highlight the stiction of the top Ge layer.

Finite-element method (FEM) simulation is performed to investigate the heat conduction in our stiction structure and the conventional suspended structure. The center of nanowire is illuminated with a pulsed optical pumping of ~ 7 kW cm^{-2} with a spot s ize of 15 μm which is above the pump power at the lasing threshold in our experiments (~3 kW cm^{-2}). The pulse duration and repetition period are 20 ns and 100 ns, respectively. The simulation temperature is set to 83 K to mimic the condition of our experiments.

Supplementary Figures 3c and d show stationary and time-dependent FEM simulations showing temperature distributions in the two structures under the aforementioned condition. While the temperature of the suspended structure is quickly elevated up to 180 K owing to optical pumping, our stiction structure shows almost negligible temperature increase (only ~0.1 K) at the same condition, thus providing the evidence of an excellent thermal conduction in our architecture. Therefore, our unique design employing stiction plays a pivotal role for the achievement of low-threshold lasing from strained Ge by allowing superior thermal management in our structure.

Supplementary Note 4: Optical cavity design

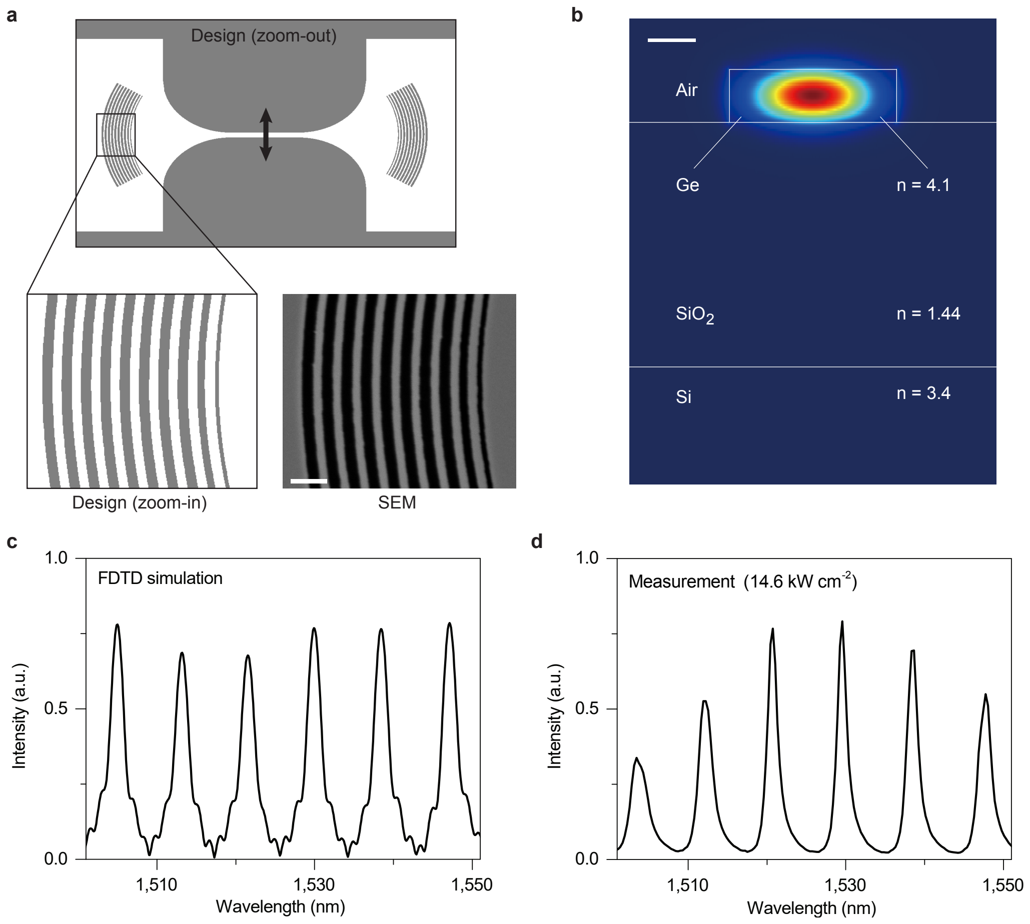

Supplementary Figure 4 | Optical cavity design. a, Design of our laser structure. A zoomed-in image and the corresponding scanning electron microscope (SEM) image of the distributed Bragg reflector (DBR) are displayed. Scale bar, 1 μm. **b,** Simulated intensity distribution of a fundamental transverse mode confined within a 700-nm-wide, 220-nm-thick germanium (Ge) nanowire lying on a silicon dioxide (SiO_2) layer. The black arrow in panel a represents the simulated region for panel b. Scale bar, 200 nm. **c,** Simulated spectrum of our structure using finite-difference time-domain (FDTD) simulation. **d,** Measured spectrum at an optical pumping density of 14.6 kW cm^{-2}.

The laser structure consists of an 8-μm long, 700-nm wide germanium (Ge) nanowire surrounded by two stressing pads containing a pair of distributed Bragg reflector (DBR) mirrors (Supplementary Fig. 4a). The DBR is carefully designed to maintain the homogeneity in the strain distribution along the nanowire. The DBR contains 10 air

trenches with width and period of 189 nm and 379 nm, respectively. The width of the first air trench (narrowed to 65 nm) and the curvature of circular arcs are optimized to obtain a high optical *Q* factor. The scanning electron microscope (SEM) image of the DBR section of the fabricated device is almost identical to the original design.

Supplementary Figure 4b shows that a fundamental transverse mode can be confined within a Ge nanowire with cross-sectional dimensions of 700 nm × 220 nm (width × height). The simulated wavelength is set to 1530 nm which is the peak wavelength of our lasing spectrum. The material stack of our device used in the simulation consists of Ge (220 nm), SiO_2 (1000 nm), and Si with refractive indices of 4.1, 1.44 and 3.4, respectively, which corresponds to the material stack used in our experiments.

Supplementary Figure 4c presents a simulated spectrum of cavity modes in 1500-1550 nm with a mode spacing of ~8 nm which is in a good agreement with our experimental result (Supplementary Fig. 4d). While the simulated *Q* factors are well above 1,000, the experimental *Q* factor at the threshold is measured to be ~850 and this discrepancy may be attributed to the sidewall roughness of our DBRs introduced during fabrication processes[8]. We believe that improving the cavity design and fabrication processes will allow us to further reduce the lasing threshold.

Supplementary Note 5: Curve fitting of experimental data

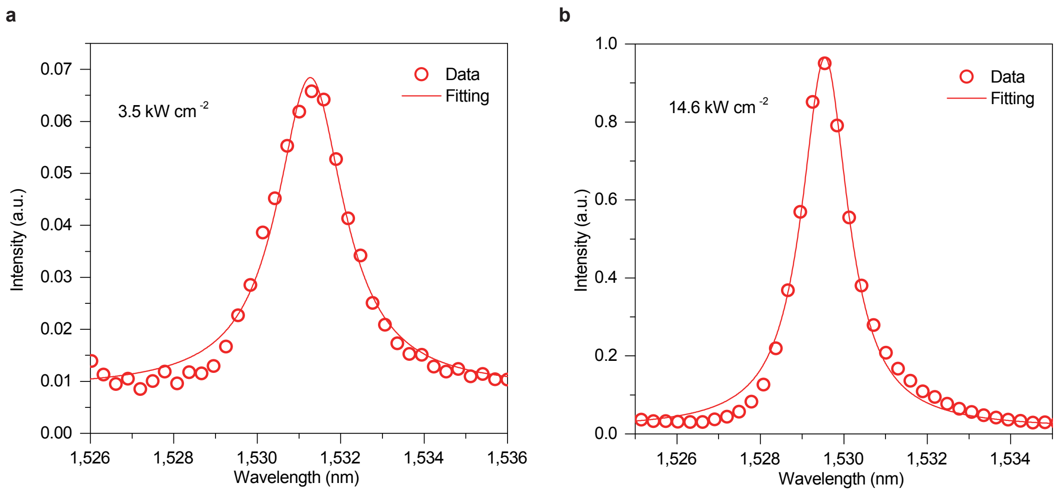

Supplementary Figure 5 | Curve fitting of lasing modes. a, High-resolution spectrum around the threshold (3.5 kW cm^{-2}). **b**, High-resolution spectrum in lasing oscillation regime (14.6 kW cm^{-2}). The red empty circles are the experimental data, and the red solid line represents the Lorentzian fitting to the experimental data.

High-resolution spectrum around the threshold (3.5 kW cm^{-2}) and in lasing oscillation regime (14.6 kW cm^{-2}) along with Lorentzian fitting results are presented in panel a and panel b, respectively. The red empty circles and the red solid line represents the experimental data and its fitting by using Lorentzian functions, respectively. The χ^2 for panel a and panel b are 3.6×10^{-4} and 2.3×10^{-2}, respectively, which give the confidence to our fitting result.

Supplementary Note 6: Multimode rate equation analysis

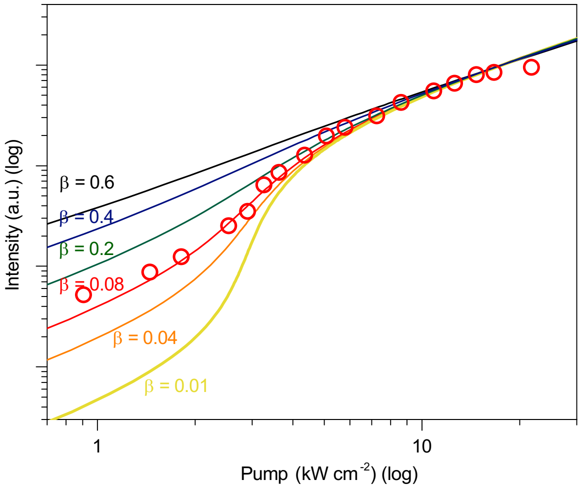

Supplementary Figure 6. Rate equation analysis. Symbols and the red line represent the measured data and the calculated theoretical curve, respectively. A spontaneous emission coupling factor, *β,* of 0.08 is extracted. The curves for different *β* values are also presented for comparison.

To compute the spontaneous emission coupling factor *β* of our germanium (Ge) nanowire, we employed the multimode laser rate equation to fit our experimental data, and obtained a *β* factor of 0.08. We assumed that the lasing modes (5 cavity modes between 1512 nm and 1547 nm) have the same threshold modal gain, group velocity and spontaneous emission factor[9]. The simplified rate equations describing the dynamic relation between carrier density and photon density is expressed as:

$$\frac{dN}{dt}=\eta_{eff}P-\frac{N}{\tau_r}-\frac{N}{\tau_{nr}}-CN^3-m_s v_g g(N)S \quad (1)$$

$$\frac{dS}{dt}=\Gamma v_g(g(N)-g_{th})S+\Gamma\beta\frac{N}{\tau_r} \quad (2)$$

where N is the carrier density in the Ge nanowire, S is the photon density, P is the optical pumping power density, η_{eff} represents the fraction of the optical pumping power absorbed by the nanowire, τ_r and τ_{nr} are the radiative and non-radiative recombination lifetimes, respectively, the Auger recombination coefficient is given as C and the number of the lasing modes is represented by m_s, v_g is the group velocity, $g(N)$ is the material

gain, g_{th} is the threshold gain, Γ is the confinement factor, and β is the spontaneous emission factor.

The fitting parameters are listed in Supplementary Table 1. The non-radiative carrier lifetime is dependent on the threading dislocation density as well as on doping concentration. The carrier lifetime of ~3.12 ns for an n-type doping density of ~1 x 10^{19} cm^{-3} has been experimentally measured in a Ge-on-insulator (GOI) sample[10]. For the specific epitaxially grown Ge layer measured for ~3.12 ns carrier lifetime, the authors used a multiple hydrogen-annealing heteroepitaxy (MHAH) growth technique. The reported threading dislocation density in the Ge layer using MHAH technique[11] ranges between 1 x 10^7 cm^{-2} ~ 1 x 10^8 cm^{-2}. In our experiment, on the other hand, we employed an As-doped Ge seed layer[2] to reduce the threading dislocation density in the Ge layer to ~4.6 x 10^6 cm^{-2}.

Previously, the carrier lifetime has been empirically correlated to the threading dislocation density as in the following equation[12]:

$$\tau = \frac{C}{\rho_D} \tag{3}$$

where τ is the minority carrier lifetime, C is a proportionality constant, ρ_D is the threading dislocation density.

To make a conservative estimation of the carrier lifetime, we here take the lower bound value of the threading dislocation density 1 x 10^7 cm^{-2} for Ge grown by MHAH technique, and take the threading dislocation density of 4.6 x 10^6 cm^{-2} for the Ge layer in our present study. Since the lifetime is inversely proportional to the threading dislocation as in Supplementary Equation 3, we estimate the lifetime of our Ge layer to be ~6.78 ns by taking into account (at least) 0.46 times lower threading dislocation density in our Ge layer compared to the Ge layer reporting 3.12 ns lifetime.

In addition, lifetime is also highly dependent on doping density as extrinsic dopants act as recombination centers. For a doping density of >10^{16} cm^{-3}, E. Gaubas *et al.*[13] showed that the carrier lifetime is related to the doping density as in the following equation:

$$\tau = \frac{1}{n_{dop}} \tag{4}$$

where n_{dop} is the doping concentration. Since we employed a doping density of 6×10^{18} cm^{-3} which is 0.6 times lower than the Ge layer with a doping density of 1 x 10^{19} cm^{-3} which presents 3.12 ns, we finally estimate the lifetime in our Ge layer to be ~11 ns. Although low temperature operation may also increase the lifetime quite as shown in ref 14, we did not include this temperature effect to be conservative in our lifetime estimation.

It is worth mentioning that the lifetime of ~11 ns is an order of magnitude less than commonly used[15] and also that the lifetime in bulk Ge with a doping density of 1 x 10^{19} cm^{-3} at our measurement temperature (~83 K) can be as large as ~600 ns[14].

For the radiative recombination lifetime, we use a direct bandgap recombination coefficient of 1.3×10^{-10} $cm^3 s^{-1}$. By calculating the fraction of the electrons residing in the direct Γ valley at a carrier injection density of 8×10^{19} cm^{-3}, we obtain 28 ns for the radiative recombination lifetime. The Auger recombination coefficient is 1 x 10^{-32} $cm^6 s^{-1}$ for the heavily doped n-type Ge.

A linear relationship between the material gain and the carrier density is assumed, which can be expressed as $g(N) = \alpha(N - N_0)$. The differential gain α of ~1.4×10^{-17} cm^2 and the transparency carrier density N_0 of ~5. 9×10^{19} cm^{-3} at the experimental gain peak wavelength of ~1530 nm are used according to our gain modelling (Supplementary Note 8).

The threshold gain is estimated from the threshold modal gain equation. The equation is expressed as:

$$\Gamma g_{th} = k_0 n_g / Q \tag{5}$$

$$k_0 = 2\pi / \lambda \tag{6}$$

where Γ is the confinement factor, Γg_{th} is the threshold modal gain at threshold, n_g is the group index, k_0 is angular wavenumber and Q is the quality factor at threshold. The confinement factor and group index are estimated from our finite-difference time-domain (FDTD) simulation: Γ = 0.45, n_g ~ 3.2. Near the threshold pumping density, the peak at 1529.6 nm has a FWHM of 1.80 nm, fitted to a Lorentzian function. The estimated Q factor at threshold is 850, and the corresponding threshold modal gain Γg_{th} is calculated to be 151.2 cm^{-1}. Thus, we estimated the threshold gain g_{th} to be 151.2/0.45 = 336 cm^{-1}.

By solving the coupled multimode laser rate equations under steady-state, we found the best fit for β is ~0.08 (plotted in Supplementary Fig. 6) and determined the lasing threshold to be ~ 3.0 kW cm^{-2}. The L-L curve for different β values was also plotted for comparison.

Supplementary Table 1. Definitions and values of the parameters for the rate equation analysis.

Parameter	Definition	Value
τ_r	Radiative lifetime	28 ns
τ_{nr}	Non-radiative lifetime	11 ns
C	Auger recombination coefficient	1×10^{-32} cm^6 s^{-1}
Q	Quality factor of the cavity	850
Γ	Optical confinement factor	0.45
m_s	Number of lasing modes	5
n_g	Group index	3.2
v_g	Group velocity ($v_g = c/n_g$)	9.38×10^9 cm s^{-1}
α	Differential gain	1.4×10^{-17} cm^2
N_0	Transparency carrier density	5.9×10^{19} cm^{-3}
Γg_{th}	Threshold modal gain	151.2 cm^{-1}
g_{th}	Threshold gain	336 cm^{-1}
β	Spontaneous emission factor	0.08

Supplementary Note 7: Strain-dependent optical characterization

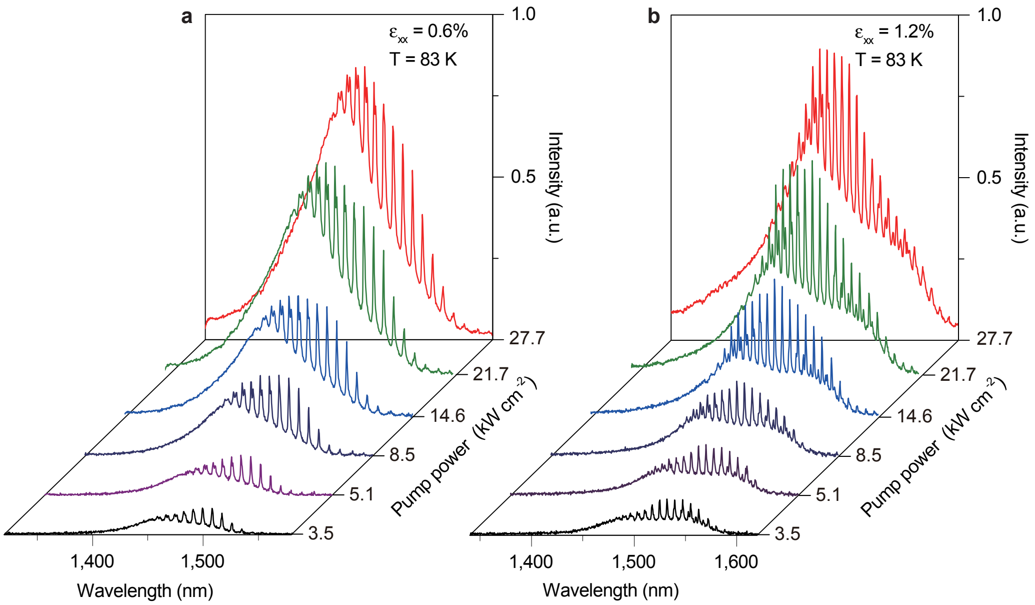

Supplementary Figure 7 | Strain-dependent optical characterization. a, Power-dependent emission spectra of a 0.6%-strained germanium (Ge) nanowire. **b**, Power-dependent emission spectra of a 1.2%-strained Ge nanowire. The amplitudes of observed resonances are smaller or comparable to the background emission due to the absence of optical net gain.

Supplementary Figure 7 presents additional spectra taken from 0.6% and 1.2% strained wires to show strain-dependent optical characteristics. While the 1.6% strained wire exhibits a clear threshold behavior as shown in Supplementary Fig. 2b of the main manuscript, 0% (shown in Fig. 2e), 0.6%, and 1.2% strained wires, on the other hand, do not show any lasing behaviour due to the insufficient strain in germanium (Ge), resulting in the absence of optical net gain. Although we observe cavity resonances from 0%, 0.6%, and 1.2% strained wires, the amplitudes of observed resonances are smaller or comparable to the amplitudes of background emission. At higher pump powers, the resonances in all low strained wires show clear saturation owing to the rapidly increasing loss.

Similar emission behaviors were observed by Kurdi *et al.*[5], where resonances were seen in a strained Ge microdisk at a low pumping level, but the ratio of the resonance

amplitude to the background emission amplitude ceased to increase as the pump power was further increased. It is clearly stated in their manuscript that in an optical cavity with a weak value of spontaneous emission coupling factor, the cavity resonances are expected to predominate over the background when lasing action occurs. Since the resonance amplitude was not overwhelming the background emission in ref 5, they did not correlate their observation to lasing as in our low strained Ge nanowires.

In stark contrast, our 1.6% strained wires (Fig. 2) show predominant resonances whose amplitudes are more than one order higher than the background emission as well as the cavity resonances outside the gain bandwidth as clearly stated in our main manuscript. And this is the result of strong superlinear increase of resonance amplitudes due to the presence of optical net gain, thus presenting clear evidence of lasing.

Our additional experiments on strain-dependent emission characteristic clearly present the pivotal role of uniaxial strain for achieving low threshold lasing in Ge by showing that the resonances become larger with regards to the background emission at higher strain, and finally overwhelm the background emission at 1.6% uniaxial strain when lasing is achieved.

Supplementary Note 8: Gain and loss modeling

Detailed description on gain and loss modeling

We perform theoretical modeling to obtain the gain spectrum of uniaxial tensile strained germanium (Ge). Empirical pseudopotential method (EPM) is used to compute the bandstructure of strained Ge. EPM is an attractive approach because it allows for the computation of bandstructures with relatively small number of empirical parameters. In the calculation for bandstructure, the single electron Hamiltonian is expressed as[16]:

$$H(G,G') = -\frac{\hbar^2}{2m}\nabla^2 + V_{loc}(|G-G'|) + V_{nloc}(G,G') + V_{so}(G,G'), \tag{7}$$

where V_{loc}, V_{nloc}, and V_{so} represent the local, nonlocal, and spin orbit contributions to the pseudopotential, respectively.

After calculating the bandstructures, the transition rates between different bands in Ge are calculated by using Fermi's golden rule. The absorption (or gain) due to band-to-band transitions is calculated using:

$$\alpha = C_0 \sum_k \delta(E_1(k) - E_2(k) - \hbar\omega)(f_1 - f_2)\,|\,p_{12}\cdot\hat{e}\,|^2, \tag{8}$$

where $\delta(E_1(k)\text{-}E_2(k)\text{-}\hbar\omega)$ computes the joint density of states (JDOS) between bands 1 and 2 corresponding to transitions energy $\hbar\omega$. $(f_1\text{-}f_2)$ represents the Fermi inversion factor where f_1 and f_2 are Fermi functions. The quantity $|\mathbf{p_{12}}\cdot\hat{e}|^2$ is the squared momentum matrix element for a particular wave vector k and $\hat{e}$ is the polarization. $C_0=\pi e^2/(m^2\varepsilon_0 cn)$ is a constant, where e is the electron charge, ε_0 is the vacuum permittivity, c is the light speed in vacuum, and n is the refractive index. The three quantities – JDOS, Fermi inversion factor and momentum matrix elements are calculated at each point in the first Brillouin zone (FBZ) and their product is then summed throughout the FBZ to obtain the material absorption (or the material gain) spectrum. For all gain curves in the main article and Supplementary Information, we only consider one polarization along the perpendicular direction to the nanowire axis since we observe the optical amplification only for such a polarization direction.

To achieve the optical net gain in Ge, the material gain should overcome the combined material loss consisting of free electron absorption (FEA) and inter-valence

band absorption (IVBA)[3]. For the losses at room temperature, we take the same formalism used in ref 3, but divide the total loss by a factor of 3 assuming isotropic losses since we only consider one polarization direction for the material gain. It is required to investigate the polarization dependence of material losses to more precisely understand the gain and loss behaviour in Ge. Since IVBA is strongly dependent on temperature[17], we take an experimentally measured IVBA at 95 K which is close to the temperature at which we observed lasing. By fitting IVBA data of Ge with p-type doping concentration of 1×10^{19} cm^{-3} at 95 K[17], we obtain:

$$\alpha_{IVBA}(\hbar\omega) = 1.13\times10^{-14} N_{h,tot} e^{-8.786(\hbar\omega)}, \tag{9}$$

where $\hbar\omega$ is the photon energy, $N_{h,tot}$ is the total hole density. α_{IVBA} is in units of cm^{-1}, $\hbar\omega$ is in units of eV, and $N_{h,tot}$ is in units of cm^{-3}. The curve for 95 K is more strongly dependent on temperature than the counterpart for 300 K, and this can be attributed to the difference in Fermi-Dirac functions at two different temperatures. We use this experimental IVBA for our modeling at 83 K, and for FEA, we employ the same formalism used for 300 K[3] since FEA is not a strong function of temperature.

Modelling results

Supplementary Figure 8a shows the separate contributions of FEA and IVBA to the combined loss for a carrier injection density of 7×10^{19} cm^{-3}. At 7 x 10^{19} cm^{-3} carrier density and at the experimental peak gain wavelength of ~1530 nm, IVBA is >214 cm^{-1} whereas FEA is only <96 cm^{-1}. Therefore, IVBA contributes to the major part of the material loss and it is critical to minimize the IVBA by lowering operating temperatures to achieve optical net gain.

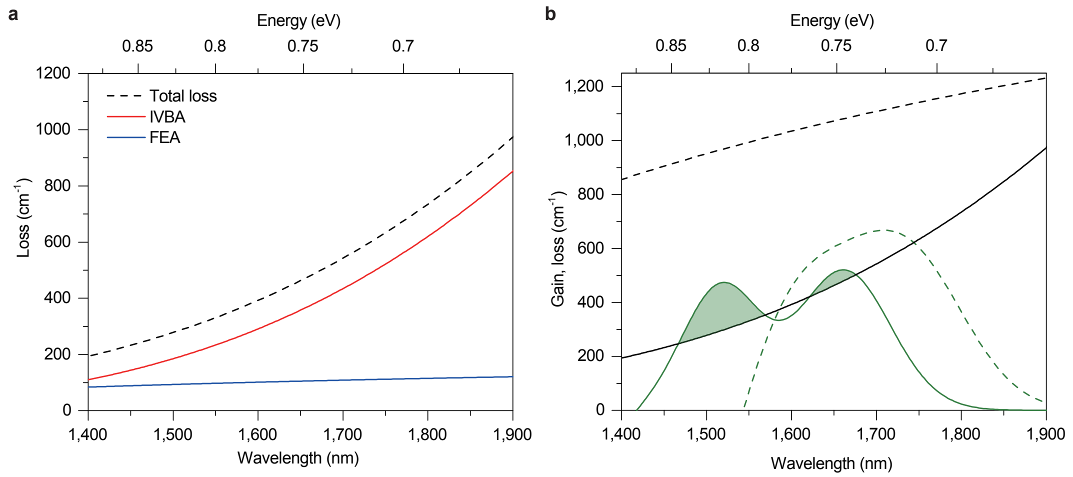

Supplementary Figure 8 | Gain and loss modelling. a, Free electron absorption (FEA) and inter-valence band absorption (IVBA) contributions to the total loss for a carrier injection density of 7×10^{19} cm^{-3}. **b,** Calculated gain (green) and loss (black) at 83 K (solid) and 300 K (dashed) for 1.6% strained Ge at a carrier injection density of 7×10^{19} cm^{-3}. The green shaded area corresponds to the positive net gain region.

Supplementary Figure 8b shows the calculated gain (green lines) and loss (black lines) at two different temperatures of 83 K (solid lines) and 300 K (dashed lines) for 1.6% strained Ge. The carrier injection density and the doping density are 7×10^{19} cm^{-3} and 6×10^{18} cm^{-3}, respectively. While the magnitudes of the peak material gain at two temperatures do not show a significant difference, IVBA can be greatly reduced at 83 K, thereby allowing the achievement of a net gain of ~180 cm^{-1}.

Supplementary Note 9: One-dimensional spatial profiles of the emission

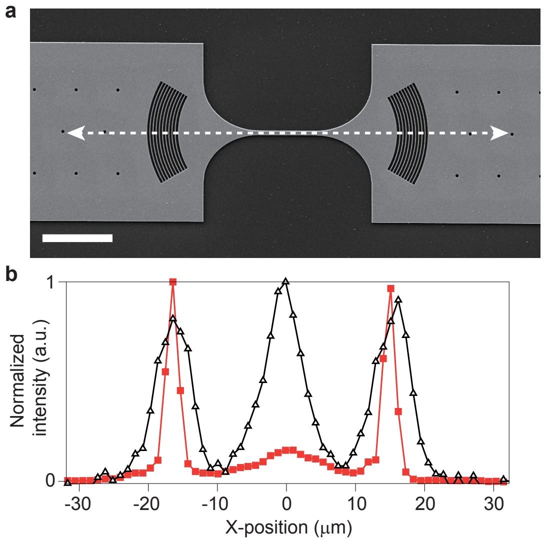

Supplementary Figure 9 | One-dimensional spatial profile of the emission. a, Scanning electron microscope (SEM) image of a typical Ge laser structure. The white dashed arrow indicates the length for which one-dimensional spatial profiles of the emission are measured. Scale bar, 10 μm. **b,** normalized one-dimensional spatial profiles of the emission for the pump powers below (black, ~1.15 kW cm^{-2}) and above (red, ~11.5 kW cm^{-2}) the lasing threshold (~3 kW cm^{-2}).

Supplemenaty Figure 9a shows a scanning electron microscope (SEM) image of a typical laser structure under 1.6% uniaxial tensile strain. The corresponding one-dimensional spatial profiles of the emission along the white dashed arrow are measured by using an InGaAs 1D-array detector as shown in Supplementary Figure 9b. For the pump power below the lasing threshold (black), the intensity of the unguided emission from the strained germanium (Ge) nanowire at the center is higher than the guided emission scattered at the distributed Bragg reflector (DBR) mirrors. For the pump power above the lasing threshold (red), the intensity of the guided emission from the DBR mirrors rapidly grows owing to the optical amplification while the linewidth of the emission beams at the DBR mirrors is significantly reduced, thus indicating the spatial coherence of the emission.

Supplementary Reference

1. Minamisawa, R. A. *et al.* Top-down fabricated silicon nanowires under tensile elastic strain up to 4.5%. *Nat. Commun.* **3,** 1–6 (2012).
2. Lee, K. H. *et al.* Reduction of threading dislocation density in Ge/Si using a heavily As-doped Ge seed layer. *AIP Adv.* **6,** 25028 (2016).
3. Suess, M. J. *et al.* Analysis of enhanced light emission from highly strained germanium microbridges. *Nat. Photonics* **7,** 466–472 (2013).
4. Nam, D. *et al.* Strain-induced pseudoheterostructure nanowires confining carriers at room temperature with nanoscale-tunable band profiles. *Nano Lett.* **13,** 3118–3123 (2013).
5. El Kurdi, M. *et al.* Tensile-strained germanium microdisks with circular Bragg reflectors. *Appl. Phys. Lett.* **108,** 91103 (2016).
6. El Kurdi, M. *et al.* Direct band gap germanium microdisks obtained with silicon nitride stressor layers. *ACS Photonics* **3,** 443–448 (2016).
7. Geiger, R. Direct band gap germanium for Si-compatible lasing. PhD Dissertation, ETH Zürich (2016).
8. Ellis, B. *et al.* Ultralow-threshold electrically pumped quantum-dot photonic-crystal nanocavity laser. *Nat. Photonics* **5,** 297–300 (2011).
9. Saxena, D. *et al.* Design and room-temperature operation of GaAs/AlGaAs multiple quantum well nanowire lasers. *Nano Lett.* **16,** 5080–5086 (2016).
10. Nam, D., Kang, J.-H., Brongersma, M. L. & Saraswat, K. C. Observation of improved minority carrier lifetimes in high-quality Ge-on-insulator using time-resolved photoluminescence. *Opt. Lett.* **39,** 6205 (2014).
11. Jain, J. R. *et al.* Tensile-strained germanium-on-insulator substrate fabrication for silicon-compatible optoelectronics. *Opt. Mater. Express* **1,** 1121–1126 (2011).
12. Sheng, J. J. *et al.* Empirical correlation for minority carrier lifetime to defect density profile in germanium on silicon grown by nanoscale interfacial engineering. *J. Vac. Sci. Technol. B Microelectron. Nanom. Struct.* **31,** 51201 (2013).
13. Gaubas, E., Bauža, M., Uleckas, a. & Vanhellemont, J. Carrier lifetime studies in

Ge using microwave and infrared light techniques. *Mater. Sci. Semicond. Process.* **9,** 781–787 (2006).

14. Camacho-Aguileria, R. E. Ge-on-Si laser for silicon photonics. PhD Dissertation, MIT (2013).

15. Liu, J. *et al.* Tensile-strained, n-type Ge as a gain medium for monolithic laser integration on Si. *Opt. Express* **15,** 11272–7 (2007).

16. Gupta, S., Magyari-Köpe, B., Nishi, Y. & Saraswat, K. C. Achieving direct band gap in germanium through integration of Sn alloying and external strain. *J. Appl. Phys.* **113,** 73707 (2013).

17. Morozov, I. & Ukhanov, I. Effect of doping on the absorption spectra of p-Ge. *Sov. Phys. J.* **13,** 744–747 (1970).
